# Attention-deficit hyperactivity disorder in people with intellectual disability: statistical approach to developing a bespoke screening tool

**DOI:** 10.1192/bjo.2021.1023

**Published:** 2021-10-04

**Authors:** Indermeet Sawhney, Bhathika Perera, Paul Bassett, Asif Zia, Regi T Alexander, Rohit Shankar

**Affiliations:** Adult learning disability services, Hertfordshire Partnership University NHS Foundation Trust, UK; Adult learning disability services, Barnet Enfield and Haringey Mental Health NHS Trust, UK; Statsconsultancy Ltd., UK; Adult learning disability services, Hertfordshire Partnership University NHS Foundation Trust, UK; Adult learning disability services, Hertfordshire Partnership University NHS Foundation Trust, UK; and School of Life and Medical Sciences, University of Hertfordshire, UK; Adult learning disabilities service, Cornwall Intellectual Disability Equitable Research (CIDER), University of Plymouth Medical School, UK

**Keywords:** Attention-deficit hyperactivity disorders, comorbidity, developmental disorders, intellectual disability, statistical methodology

## Abstract

**Background:**

Attention-deficit hyperactivity disorder (ADHD) is common among people with intellectual disability. Diagnosing ADHD in this clinically and cognitively complex and diverse group is difficult, given the overlapping psychiatric and behavioural presentations. Underdiagnoses and misdiagnoses leading to irrational polypharmacy and worse health and social outcomes are common. Diagnostic interviews exist, but are cumbersome and not in regular clinical use.

**Aims:**

We aimed to develop a screening tool to help identify people with intellectual disability and ADHD.

**Method:**

A prospective cross-sectional study, using STROBE guidance, invited all carers of people with intellectual disability aged 18–50 years open to the review of the psychiatric team in a single UK intellectual disability service (catchment population: 150 000). A ten-item questionnaire based on the DSM-V ADHD criteria was circulated. All respondents’ baseline clinical characteristics were recorded, and the DIVA-5-ID was administered blinded to the individual questionnaire result. Fisher exact and multiple logistic regressions were conducted to identify relevant questionnaire items and the combinations that afforded best sensitivity and specificity for predicting ADHD.

**Results:**

Of 78 people invited, 39 responded (26 men, 13 women), of whom 30 had moderate-to-profound intellectual disability and 38 had associated comorbidities and on were medication, including 22 on psychotropics. Thirty-six screened positive for ADHD, and 24 were diagnosed (16 men, eight women). Analysis showed two positive responses on three specific questions to have 88% sensitivity and 87% specificity, and be the best predictor of ADHD.

**Conclusions:**

The three-question screening is an important development for identifying ADHD in people with intellectual disability. It needs larger-scale replication to generate generalisable results.

Attention-deficit hyperactivity disorder (ADHD) is a neurodevelopmental disorder characterised by a persistent pattern of inattention and/or hyperactivity and impulsivity, with an onset in childhood, and causes significant functional impairment to the individual.^1^ The prevalence rate is 5.9% in youth and 2.5% in adults.^2^ It is estimated to be much higher among people with intellectual disability.^3^ Some studies have estimated a prevalence of around 20%.^4^ ADHD causes significant functional impairment in physical, mental and social well-being.^2,5^ Treatment of ADHD has shown to improve long-term outcomes such as obesity, non-medicinal drug use/addictive behaviour, antisocial behaviour, use of health services, self-esteem and social function outcomes.^2,6^

Although there is a higher prevalence of ADHD in people with intellectual disability, ADHD continues to remain underdiagnosed and misdiagnosed in this population.^7,8^ Various reasons can be hypothesised as contributory factors for this underdiagnosis (Table 1). Diagnostic overshadowing particularly of misdiagnosing ‘challenging behaviour’ can also be a manifestation of underlying unrecognised ADHD in people with intellectual disability.^9^ Effective treatment of ADHD has a positive effect on symptom control and reduction of the core features of ADHD, as well as quality of life and daily functioning.^9,10,11^ People with intellectual disability and ADHD are less likely to use antipsychotic medications compared with their peers who are not receiving ADHD treatment.^9,10,12^ Hence, it is imperative that ADHD is identified as early as possible, and managed appropriately.

**Table 1:** Complexity contributing to underdiagnoses and misdiagnosis of ADHD in people with intellectual disability

Challenges in diagnosing ADHD in intellectual disability
Patient factors
Communication difficulties limit the person's ability explain ADHD symptoms
High neurodevelopmental comorbidities, such as autism spectrum disorder, affecting/masking symptoms of ADHD
High prevalence rate of mental illness affecting/masking symptoms of ADHD
Effect of ADHD symptoms may be limited because of the person's level of intellectual disability
Difficulty defining whether inattention and hyperactivity symptoms are attributable to intellectual disability or ADHD
ADHD may be manifested as challenging behaviour
Clinical factors
Difficulties obtaining a developmental history
Difficulties in defining functional impairment
Several DSM-V ADHD criteria are not applicable for people with intellectual disability and communication difficulties
Limited published research in ADHD in intellectual disability
Lack of awareness and training in diagnosis and treatment of ADHD in intellectual disability
Ambivalence about the existence of ADHD in intellectual disability

ADHD, attention-deficit hyperactivity disorder.

The Diagnostic Interview for ADHD in adults with intellectual disability (DIVA-5-ID) is a diagnostic tool recommended for use in people with intellectual disability.^13^ Using the DIVA-5-ID requires significant resources and time. For the general population, validated screening tools can be used before using the DIVA-5.^14^ However, for people with intellectual disability, there is no such validated screening tool. The screening tools that are currently available from general population are of limited use in people with intellectual disability.^12^ They were developed in general population, making their routine application in people with intellectual disability problematic without further testing. In addition, the predictive validity of these tools is invariably poorer when used outside of the sample in which they were developed. Further, there is no consensus on which outcomes are the most important.^15^ This paper seeks to provide an evidence-based concept for a screening tool specific for people with intellectual disability and ADHD.

## Aim

We aimed to identify the predictive ability of specific symptoms of ADHD as applied to people with intellectual disability, to develop a screening tool.

## Method

### Study design and development sample

This is a cross-sectional study undertaken in January 2019, using the Strengthening the Reporting of Observational Studies in Epidemiology (STROBE) guidance. The study population was people with intellectual disability aged 18–50 years, open to review of the psychiatric team of a specialist National Health Service (NHS) intellectual disability service in the UK, covering a catchment of 152 000 people. Those aged >50 years or with a diagnosis of dementia were not included.

The service was developing pathways for various neurodevelopmental conditions and was using screening tools developed by clinicians as part of routine practice. For ADHD, a ten-item questionnaire was developed based on the current DSM-V ADHD diagnostic criteria, with an additional question relating to challenging behaviour (Appendix 1).^16^

The ten items were chosen based on a previous study highlighting that certain items used in the process of diagnosing ADHD in general population (i.e. ‘loosing things’, ‘forgetful in daily activities’, ‘talking excessively’, ‘often blurt out answers’ and ‘interrupt or intrude on others’) were not reliable in people with intellectual disability.^17^ The items that were left out relied on the patient possessing a higher level of functioning.

The recruitment process is provided in Appendix 2. The questionnaire was sent to the carers of people with intellectual disability, with instructions to be completed by a person who knew the patient well. The service clinicians assessed all of the patients whose carers replied to the questionnaire, using the DIVA- 5-ID, before reviewing the questionnaire. If one or more questions on the questionnaire were answered ‘yes’, it was considered a positive screening. Comparisons were then made between DIVA-5-ID and the questionnaire results. Baseline patient characteristics of all respondents, including gender, age, level of intellectual disability, number and type of comorbidities, and prescribed psychotropic medication was collected.

### Ethics/governance

The project was done with anonymised data from a single centre. No patient data was shared outside of the clinical team. Data were collected as part of ongoing service evaluation, formally registered with the host NHS organisation. The NHS Health Research Authority tool (http://www.hra-decisiontools.org.uk/research/index.html) was used to confirm that no ethics approval was needed for this project (Supplementary File 1 available at https://doi.org/10.1192/bjo.2021.1023). No author had access to any patient-identifiable information other than that of their own clients in their service. All survey recipients had been informed of the reasons for the survey, that consent was given via return of the survey, that data would be used for both clinical and research use and all data would be anonymised before sharing outside of the clinical team.

### Statistical analysis

A specialist statistician outlined a four-stage approach to identify the reliability of the questionnaire questions compared with the gold-standard DIVA-5-ID.

#### Stage 1

The separate association between each of the ten screening questions and the DIVA-5-ID results across all participants was examined with Fisher's exact test.

#### Stage 2

The DIVA-5-ID result was used as the outcome variable, with the screening questions as the predictor variables, using multiple logistic regression. To simplify the regression model, variables not found to be associated with the outcome were omitted from the model. This was done with a backward-selection approach, with the question with the largest *P*-value removed at each point. It was recognised that because of the relatively small sample size, questions could be associated with the DIVA-5-ID result, but not achieve statistical significance. Therefore, two different approaches were considered, resulting in two different models. The first model (model 1) included all variables where the odds ratio was >2. A second approach (model 2) only included questions in the final model if the *P*-value was <0.2. For each variable in the two models, results are presented as odds ratios for each question, with their corresponding confidence intervals. These give the odds of a positive DIVA-5-ID result when the answer was ‘yes’ compared with odds when the answer was ‘no’.

#### Stage 3

The next stage aimed to determine a score based on the screening questions that could best be used to predict the DIVA-5-ID result. The regression coefficients for each question were calculated. A rule was established that for any question found to be significant, they would be multiplied by their effect size based on their individual regression coefficients.

- Score 1: All questionnaire questions with >20% difference in positive DIVA-5-ID rates between responses, regardless of statistical significance.
- Score 2: Questionnaire questions with an odds ratio of >2 from the multiple logistic regression analysis, regardless of statistical significance.
- Score 3: Questionnaire questions with a *P*-value of <0.2 from the multiple logistic model.
- Score 4: As score 3, but giving more weight to questions more associated with the DIVA-5-ID result.

#### Stage 4

The final analyses examined the ability of the collective scores (1–4) to predict the DIVA-5-ID result. Receiver operating characteristic (ROC) curves were used to examine the predictive ability, with the area under the ROC curve quantifying the performance. The ROC curves were used to choose an optimal cut-off point for each score that could best predict the DIVA-5-ID result. The cut-off point was chosen so as to give the best combination of sensitivity and specificity. The positive and negative predictive values and diagnostic odds ratio have also been showcased.

## Results

The recruitment numbers by stage are outlined in Appendix 2. There were 78 eligible people open to the psychiatric team. Of the 78 questionnaires sent out, 39 (50%) were returned; 26 male (66%) and 13 female (33%) respondents, with a mean age of 38 years. Thirty had moderate-to-profound intellectual disability (77%) and nine had mild intellectual disability (33%). Only one person did not have a comorbid health issue. Of the 38 respondents with comorbid health issues, 14 had one (36%), 14 had two (36%) and ten had three or more (26%) comorbid conditions. Specific psychiatric comorbidities and the number of patients with the diagnosis are provided in Table 2.

**Table 2:** Various psychiatric diagnosis and psychotropics/antiepileptic drugs prescribed

	Number of patients
Different psychiatric comorbidities	
Autism	19
Anxiety	14
Epilepsy	10
Mood disorders	8
Psychosis	1
Different psychotropic and antiepileptic medication	
Lamotrigine (AED/mood stabilizer)	7
Levetiracetam (AED)	3
Risperidone (antipsychotic)	15
Epilim chrono (AED)	3
Carbamazepine (AED)	7
Sertraline (SSRI)	5
Fluoxetine (SSRI)	2
Citalopram (SSRI)	7
Aripiprazole (antipsychotic)	2
Mirtazapine (SNRI)	3
Quetiapine (antipsychotic)	2
Olanzapine (antipsychotic)	3
Depakote (mood stabiliser)	1
PRN zopiclone (sedative/hypnotic)	1
PRN lorazepam or diazepam (benzodiazepine sedative/hypnotic/anxiolytic)	18

AED, antiepileptic drug; SSRI, Selective Serotonin Reuptake Inhibitor; SNRI, Serotonin and norepinephrine reuptake inhibitor; PRN, pro re nata.

Just one person was not on any medication. Seven people were on one medication, 15 were on two medications and 16 were on three or more medications. Looking specifically at psychotropics, 22 people were prescribed antipsychotics (56%), 17 were prescribed antidepressants (44%), 12 were prescribed anti-seizure medication (31%) and 10 were prescribed mood stabilisers (26%); 21 people were prescribed two or more psychotropics (54%). The specific drugs and number of patients prescribed are provided in Table 2.

Of the 39 respondents, 36 (92%) had answered at least one question of the questionnaire positively, with three replying negatively to all questions. The corresponding DIVA-5-ID of all 39 respondents was completed, which identified 24 (61%) as having ADHD, of whom 16 were male (67%) and eight were female (33%).

### Stage 1

Significant associations (*P* < 0.05) with the DIVA-5-ID were found for questions 1, 2, 3 and 6, and borderline significance (*P* = 0.06) for question 8 (Table 3). For the questions where a significance was seen, DIVA-5-ID responses of ‘yes’ were in, higher numbers for questions 1 and 2 where >90% answered ‘yes’ compared with less than 40% answering ‘no’.

**Table 3:** Association between individual questions and DIVA-5-ID result

Question	Category	Negative DIVA-5-ID result, *n* (%)	Positive DIVA-5-ID result, *n* (%)	*P*-value
Q1	No	13 (72%)	5 (28%)	<0.001
	Yes	2 (10%)	19 (90%)	
Q2	No	14 (64%)	8 (36%)	<0001
	Yes	1 (6%)	16 (94%)	
Q3	No	10 (63%)	6 (37%)	0.02
	Yes	5 (22%)	18 (78%)	
Q4	No	4 (50%)	4 (50%)	0.69
	Yes	11 (35%)	65 (65%)	
Q5	No	6 (55%)	5 (45%)	0.28
	Yes	9 (32%)	68 (68%)	
Q6	No	8 (73%)	3 (27%)	0.01
	Yes	7 (25%)	21 (75%)	
Q7	No	8 (57%)	6 (43%)	0.10
	Yes	7 (28%)	18 (72%)	
Q8	No	10 (56%)	8 (44%)	0.06
	Yes	5 (24%)	16 (76%)	
Q9	No	8 (53%)	7 (47%)	0.18
	Yes	7 (29%)	17 (71%)	
Q10	No	5 (57%)	4 (44%)	0.27
(2 categories)	Yes	10 (33%)	20 (67%)	
Q10	No	5 (57%)	4 (44%)	0.37
(3 categories)	Yes, one option	1 (17%)	5 (83%)	
	Yes, both options	9 (37%)	15 (63%)	

DIVA-5-ID, Diagnostic Interview for ADHD in adults with intellectual disability.

### Stage 2

The logistic regression of model 1, comprising five questions (odds ratio > 2; i.e. questions 1, 2, 3, 6 and 7), had a greater than two times odds of a positive DIVA-5-ID result with a ‘yes’ compared with a ‘no’ response (Table 4). Only question 2 was statistically significant (*P* < 0.05). As per the statistical plan, variables with larger *P*-values (≥ 0.2) were removed from model 1, leaving three questions for model 2 (i.e. questions 1, 2 and 6). Question 2 was the most associated with DIVA-5-ID result, with borderline significance (*P* = 0.05). For question 2, the odds ratio was 16 times higher for a positive DIVA-5-ID result and a ‘yes’ response compared with a ‘no’ response.

**Table 4:** Multiple logistic regression results

Model	Question	Odds ratio (95% CI)^a^	Regression coefficient	*P*-value
1	Q1	2.89 (0.29–29.2)	1.06	0.37
	Q2	22.1 (1.24–3947)	3.10	0.04
	Q3	2.05 (0.27–15.3)	0.72	0.49
	Q6	7.61 (0.57–101)	2.03	0.12
	Q7	2.72 (0.32–23.4)	1.00	0.36
2	Q1	5.32 (0.66–43.2)	1.67	0.12
	Q2	16.5 (1.05–257)	2.80	0.05
	Q6	6.40 (0.54–76.3)	1.86	0.14

DIVA-5-ID, Diagnostic Interview for ADHD in adults with intellectual disability.

a. Odds ratios represent the odds of a positive DIVA-5-ID result for a ‘yes’ response relative to the odds for a ‘no’ response.

### Stage 3

Each eligible question in the scores 1–4 was allotted one point for a ‘yes’ response and no points for a ‘no’ response. The exception was for score 4, where a ‘yes’ response to question 2 was allotted 2 points, as this variable was found to be most associated with a positive DIVA-5-ID score, as the regression coefficient for question 2 was approximately twice that of questions 1 and 6, which were similar. The scores were as follows, where Q indicates question:

- Score 1: Q1 + Q2 + Q3 + Q5 + Q6 + Q7 + Q8 + Q9 + Q10
- Score 2: Q1 + Q2 + Q3 + Q6 + Q7
- Score 3: Q1 + Q2 + Q6
- Score 4: Q1 + 2.Q2 + Q6.

### Stage 4

**Fig. 1:**
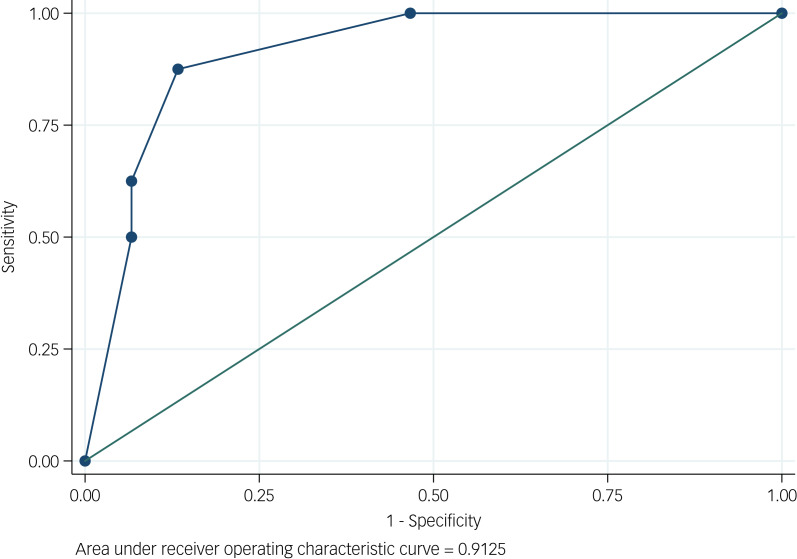
Diagnostic performance of score 4.

The diagnostic performance of each of the four scores for the prediction of the DIVA-5-ID result was evaluated (Table 5). The results suggested relatively good diagnostic performance for all four scores, with all area under the curve values being ≥0.86. Score 4 (questions 1, 2 and 6) had the highest area under the curve value (Fig. 1). This score had a range of values from 0 to 4, a sensitivity of 88%, a specificity of 87% and a score of ≥2, and was found to be the best predictor of a positive DIVA-5-ID result. All four scores had a positive predictive value of >90%, with score 2 being highest at 95% and score 4 being 91%. The negative predictive values for the four scores were dispersed (range 65–81%), with score 4 being the highest at 81%. The odds of a positive test in those with ADHD relative to the odds of a positive test in those without ADHD was highest in score 2 (70.0), with score 4 being second highest at 45.5.

**Table 5:** Performance of different scores for the prediction of DIVA-5-ID result

Score	Range values	AUC (95% CI)	Optimal cut-off point	Sensitivity	Specificity	Positive predictive value	Negative predictive value	Diagnostic odds ratio
1	0–9	0.86 (0.75–0.97)	≥6	71%	87%	90%	65%	15.8
2	0–5	0.90 (0.80–0.99)	≥3	83%	93%	95%	78%	70.0
3	0–3	0.90 (0.79–1.00)	≥2	83%	87%	91%	77%	32.5
4	0–4	0.91 (0.81–1.00)	≥2	88%	87%	91%	81%	45.5

DIVA-5-ID, Diagnostic Interview for ADHD in adults with intellectual disability; AUC, Area under the curve.

## Discussion

Diagnosing ADHD in adults with intellectual disability can be challenging, and there are many barriers to diagnosis (Table 1). Using the DIVA-5-ID as a diagnostic tool can be resource-intensive for daily clinical practice, as there is a need to collect comprehensive past and present history and interview a reliable informant, and it requires trained specialist clinical input. Thus, screening tools are of value where ADHD is suspected. However, in a population with a high prevalence of ADHD, there is no screening tool or decision-support tool to undertake a full assessment. Equally, as shown by our study sample, this group has multimorbidity and is subject to polypharmacy. This innovation in routine clinical practice is the first evidence-based attempt to develop a screening tool for ADHD in people with intellectual disability.

This tool has the potential to influence positive change in supporting the health needs of people with intellectual disability. The obvious benefit is to quickly identify people with intellectual disability with suspected ADHD for further diagnostic work-up, i.e. the DIVA-5-ID. Other benefits include reduction of misdiagnosis and, by extension, reduction in polypharmacy, particularly in the prescribing of inappropriate psychotropics in a vulnerable population. As shown, the majority of the study sample pre-screening were on psychotropic medication, with over half (54%) on multiple drugs. It could be that these drugs were prescribed to manage presenting symptoms of ADHD in the individual, without consideration of an ADHD diagnosis. This could have led to limited symptom relief. Better screening can lead to improved diagnosis, improving both health and quality of life for the patient. Furthermore, it is carer- or patient-led, thus having positive implications on clinician resource and time.

### Implications for patients and their carers/families

Because of cognitive deficits, people with intellectual disability can be marginalised in their ability to interactively participate in a clinical process. Three-quarters of the patients in this study (77%) had a moderate-to-profound disability, which would affect their meaningful contribution to the diagnostic or treatment assessments. This disadvantage can be further compounded by the presence of ADHD. In these circumstances, clinicians often have to depend on the narrative from informants. This information from family members or professional carers could potentially bring an emotional and subjective bias to the diagnostic process. The availability of a structured questionnaire allows better evidence-based synthesis to the clinical formulation about the presence or absence of ADHD. The questionnaire will allow for stepwise discussion with the patient and other stakeholders and planning, including the justification for trialling ADHD medication.

### Implications for policy and research

ADHD remains an unrecognised comorbidity in people with intellectual disability. This has, in many cases, led to overprescribing of psychotropic medication, which can lead to several unwanted effects on well-being, morbidity and mortality.^18,19^ Further development and national adoption of the screening tool could help to mitigate these adverse outcomes.

The lack of a screening tool despite the need in this vulnerable population could also be because of the heterogeneous complex presentation of this population, particularly with regards to multimorbidity, polypharmacy and intellectual disability-specific characteristics confounding the picture. The current questionnaire looks to overcome these challenges of heterogeneity by using statistical concepts, and has shown potential for further study. In particular, multi-site implementation of score 4 (questions 1, 2 and 6; bolded in the questionnaire) needs to be conducted to see if the clinical yield is similar to this pilot study. Other psychometric properties of these three questions may need further research.

Another potential area to explore would be to examine if the screening questions not only suggest ADHD, but have any discriminating validity in ruling out other reasons for ADHD symptoms, such as other serious mental illness.

This is a pragmatic single-site study carried out as part of routine clinical practice. Hence, no advance power calculations on sampling were made. However, it is worth recognising that the study was prospective and the clinician investigators were blinded to the questionnaire results. The identified questions (questions 1, 2 and 6) are predominantly focused on observed behaviour, which could also be a presentation of other medical and psychological conditions such as drug-induced akathisia, autism spectrum disorder, etc. However, it is the study premise that this is not a diagnostic tool but a screening tool. It is not suggested to replace the gold standard of inquiry into such behavioural patterns.

Score 2 (questions 1, 2, 3, 6 and 7) was the other potential alternative as it had higher specificity (93%), higher positive predictive value (95%) and a better odds ratio (70) than score 4. However, as the focus is on screening, score 4 had a better negative predictive value and better sensitivity, in addition to only being three questions (questions 1, 2 and 6). It is worth noting that these three questions also feature in score 2. In future, larger field trials, it might be worth considering a more inclusive screening set (i.e. score 2), to revisit comparisons between score 2 and score 4 as to which would be better suited.

In the statistical work-up, assumptions had to be made based on the sample size and expected associations. A *P*-value of 0.2 was taken for stage 2 instead of *P* < 0.05 or *P* < 0.10, as the data-set was small and likely to be predisposed to big associations for some questions that did not reach statistical significance. With a bigger sample, it is likely significant results would be obtained with similar effect size. The threshold here was relaxed to allow more variables into the score. It was felt that to make the scores simpler to calculate in practice, a simpler strategy of assigning points to each factor should be used. Using the exact coefficients would mean that more involved calculations would be required to calculate the score for each patient, which may limit the use of the scoring system. As outlined in the paper, the first scores used equal weighting for each variable. Approximate regression coefficients were used to give different weightings to the questions for the last of the scores.

It is possible that more people who are engaged or interested in the support of ADHD responded to the questionnaire than people who are not. Also, it was not possible to explore the characteristics of non-responders. This may have introduced bias in the data. However, the response rate of 50% suggests that this is unlikely. Also, some questions might be perceived as ambiguous and there may be some overlap between questions. Carers relying on retrospective memory and reports are likely to lead to approximations. Despite its limitations, the study has captured critical knowledge and evidence hitherto unavailable in scientific literature.

In conclusion, there is a significant level of underdiagnoses of ADHD in people with intellectual disability. This has led to misdiagnosis and polypharmacy, particularly for psychotropics. This study delivers three evidence-based screening questions to assist carers and clinicians to consider further ADHD diagnostic work-up: ‘Does he/she find hard to sit in one place for long?’, ‘Does he/she pace up and down most of the time?’ and ‘Is he/she easily distractible by busy environments?’.

These three questions could be easily incorporated into any preliminary inquiry into a referral for a psychiatric or behavioural assessment of a person with intellectual disability, to help consider ADHD and provide better clinical formulation and bespoke treatment of their needs.

## Data Availability

The data that support the findings of this study are available from the corresponding author, R.S., upon reasonable request.

## Supplementary material

For supplementary material accompanying this paper visit http://dx.doi.org/10.1192/bjo.2021.1023. click here to view supplementary material

## Appendix 1: ADHD screening questionnaire for people with intellectual disability (questions identified for final screening in bold)

**Table:**

Question		Yes/no (present in the past 6 months)	Tick if this symptom goes back to childhood
**Q1**	**Does he/she find hard to sit in one place for long?**		
**Q2**	**Does he/she pace up and down most of the time?**		
Q3	Does he/she often fidget with their hands/feet?		
Q4	Is he/she very impulsive/can't wait for his turn?		
Q5	Does he/she often intrude/interrupt others?		
**Q6**	**Is he/she easily distractible by busy environments?**		
Q7	Does he/she often move from one task/activity to another?		
Q8	Does he/she appear preoccupied and not listening when spoken to directly?		
Q9	Is he/she forgetful in daily tasks and need frequent reminders and prompting?		
Q10	Does the patient display any challenging behaviour?	If yes, please tick the following that apply (you can choose one or more)	
		• Physical aggression towards others • Damage to property	
